# Integrated Neurobiology of Bipolar Disorder

**DOI:** 10.3389/fpsyt.2014.00098

**Published:** 2014-08-25

**Authors:** Vladimir Maletic, Charles Raison

**Affiliations:** ^1^Department of Neuropsychiatry and Behavioral Sciences, University of South Carolina School of Medicine, Columbia, SC, USA; ^2^Department of Psychiatry, University of Arizona, Tucson, AZ, USA; ^3^Norton School of Family and Consumer Sciences, College of Agriculture and Life Sciences, University of Arizona, Tucson, AZ, USA

**Keywords:** bipolar disorder, neurobiology, inflammation, glial, imaging, neurotransmitters, mania, depression

## Abstract

From a neurobiological perspective there is no such thing as bipolar disorder. Rather, it is almost certainly the case that many somewhat similar, but subtly different, pathological conditions produce a disease state that we currently diagnose as bipolarity. This heterogeneity – reflected in the lack of synergy between our current diagnostic schema and our rapidly advancing scientific understanding of the condition – limits attempts to articulate an integrated perspective on bipolar disorder. However, despite these challenges, scientific findings in recent years are beginning to offer a provisional “unified field theory” of the disease. This theory sees bipolar disorder as a suite of related neurodevelopmental conditions with interconnected functional abnormalities that often appear early in life and worsen over time. In addition to accelerated loss of volume in brain areas known to be essential for mood regulation and cognitive function, consistent findings have emerged at a cellular level, providing evidence that bipolar disorder is reliably associated with dysregulation of glial–neuronal interactions. Among these glial elements are microglia – the brain’s primary immune elements, which appear to be overactive in the context of bipolarity. Multiple studies now indicate that inflammation is also increased in the periphery of the body in both the depressive and manic phases of the illness, with at least some return to normality in the euthymic state. These findings are consistent with changes in the hypothalamic–pituitary–adrenal axis, which are known to drive inflammatory activation. In summary, the very fact that no single gene, pathway, or brain abnormality is likely to ever account for the condition is itself an extremely important first step in better articulating an integrated perspective on both its ontological status and pathogenesis. Whether this perspective will translate into the discovery of innumerable more homogeneous forms of bipolarity is one of the great questions facing the field and one that is likely to have profound treatment implications, given that fact that such a discovery would greatly increase our ability to individualize – and by extension, enhance – treatment.

## Introduction

Despite significant advances in our understanding of the underlying neurobiology of bipolar disorder, its timely diagnosis and efficient treatment remain daunting clinical challenges. Multiple psychiatric comorbidities, including attention deficit hyperactivity disorder (ADHD) as well as anxiety, personality, and eating and substance use disorders, interfere with diagnosis and treatment and likely contribute to increased disease morbidity and mortality in general and to increased suicide risk in particular (1, 2). In addition to an increased risk of suicide, bipolar disorder is also associated with considerable medical comorbidities, including cardio- and cerebrovascular disease, and metabolic and endocrine disorders, which, when combined with neuropsychiatric morbidity and suicidality, have been found to reduce life expectancy by an average of 11 years in females and 10 years in males afflicted with bipolarity (1, 3).

These poor outcomes reflect our growing recognition, based on neurobiological and neuroimaging research, which bipolar disorder is frequently an aggressive and corrosive condition. Epidemiologic studies suggest that repeated mood episodes and even minor, residual symptoms enhance the risk of future recurrences (4–7). Successive episodes have, in turn, produce detectable volumetric changes in the brain that have been frequently associated with deterioration in multiple functional domains (8–11). Moreover, contrary to previous views, we now know that neuropsychological deficits often persist even when individuals with the disorder are in a euthymic state (12–14).

Unfortunately, our current diagnostic schema for bipolar disorder, which is based on descriptive nomenclature rather than clearly delineated causal mechanisms, has not given rise to treatments that provide sustained, symptomatic, and functional recovery for many patients (15). Moreover, available pharmacologic interventions are plagued by pronounced adverse effects that often aggravate metabolic status and further compromise cognition in people already struggling in this domain (16–18). Finally, treatment-related adversities and polypharmacy tend to translate into sub-optimal treatment adherence (19).

Is there a way out of this vicious cycle? Fortunately, the preponderance of genetic, neuroimaging, histological, and biochemical studies provide a different perspective on bipolar disorder as a biologically diverse disease category. Greater understanding of the important pathophysiological differences between bipolar subtypes will increasingly help maximize treatment efficacy while minimizing unwanted side effects and adverse events. Taken as a whole, the current state of the science strongly suggests that rather than being a single condition, the diagnostic entity we call bipolar disorder is composed of diverse biological entities, with phenotypical manifestations similar enough to each other to fit under the same diagnostic umbrella. This reframing of bipolar disorder immediately raises questions. Does this perspective point to more advantageous ways of diagnosing and/or treating the disorder? For example, might it be that assuming an approach similar to the one used to define and treat complex medical conditions may be more fruitful than our current approaches to bipolar disorder? Do we have sufficient knowledge to characterize bipolar disorder based on its genetics, etiopathogenesis, pathophysiology, and alterations on the cellular and subcellular levels? Given the genetic and neurobiological diversity of bipolar disorder, is there a reasonable hope that we can achieve anything more than a probabilistic association between pathophysiological underpinnings and clinical manifestations of the condition?

**Figure 1:**
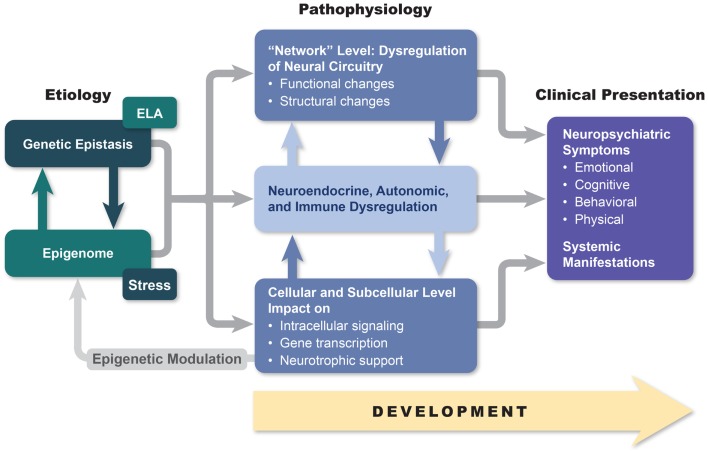
**An etiopathogenesis-based understanding of mood disorders**. Descriptive models of mood disorders offer only minimal treatment guidance. A model connecting genotype, epigenetic modification, and multiple-level endo-phenotypical alterations to clinical presentation may provide a path to greater treatment success. Our model acknowledges pathophysiological diversity of mood disorders and provides opportunity for individualized treatment approaches based on the link between symptom constellations, genetics, and specific endo-phenotype markers.

Although the answers to these questions are not known with any finality, we hope to demonstrate in this paper that a deeper understanding of the relationship between macroscopic and microscopic brain changes (including alterations in cellular and subcellular signaling) and the phenotypical manifestations of bipolar disorder may open the possibility of developing more effective and less disruptive treatment approaches. Furthermore, we believe that the brain network changes and alterations in neurotransmission that are characteristic of bipolar disorder disrupt brain–body signaling in ways that may in the future allow for novel therapeutic methodologies that reverse the autonomic, neuroendocrine, and immune systems that are characteristic of the disorder and that almost certainly contribute to the high degree of medical morbidity observed in bipolarity. Finally, we will attempt to establish a link between macroscopic and microscopic brain changes and the interaction between multiple genetic factors and life adversities (Figure 1). As a first step in realizing these aspirations, in this paper, we review genetic, neuroimaging, pathohistological, neuroendocrine, and molecular research in hope of finding answers that may be useful for helping bipolar patients in our everyday clinical practices.

## Genetic Findings in Bipolar Disorder

A strong genetic basis for bipolar disorder has been apparent since researchers conducted the first familial and identical twin studies many years ago. Identical-twin concordance rates for bipolar disorder generally range from 40 to 70%, with the estimated heritability reaching as high as 90% in the most recent reports (20). However, despite these observations, the unambiguous identification of single nucleotide (SNP) risk factors for the disorder has proven remarkably difficult. Nonetheless, the “gold standard” genome-wide association study (GWAS) approach, although initially disappointing, has begun to yield consistent SNP and genetic pathway findings for bipolar I disorder. Interestingly, however, although some difference in genetic risks have been observed between bipolar disorder and other currently recognized psychiatric disease states, the more striking finding is the high degree of genetic overlap between conditions. For example, a pronounced genetic overlap, primarily between schizophrenia and bipolar disorder, but also with major depressive disorder (MDD), has been recently reported (21). Perhaps surprisingly, and contradicting traditional diagnostic schemes, a recent GWAS suggests that bipolar disorder is genetically more closely related to schizophrenia than MDD (22). In general, GWAS suggest that both bipolar I disorder and schizophrenia are characterized by polygenic inheritance, such that many common variants, each with a very small effect size, contribute to the disorders (23, 24). These genetic risks appear not to be randomly scattered through the genome, but rather to coalesce into functional pathways. For example, a recent GWAS found evidence for enrichment of risk SNPS in the following pathways: corticotropin-releasing hormone signaling, cardiac β-adrenergic signaling, phospholipase C signaling, glutamate receptor signaling, endothelin 1 signaling, and cardiac hypertrophy signaling (25). And despite the high degree of genetic overlap between bipolar I disorder and schizophrenia, recent GWAS also indicate the existence of non-shared polygenic pathways for each condition (26, 27). Moreover, recent studies indicate that large effect size copy number variants are more common in schizophrenia, and that schizophrenia may be more closely tied to central nervous system (CNS) autoimmune processes (i.e., multiple sclerosis) than is bipolar disorder (28, 29).

Not surprisingly, genetic studies have confirmed that bipolar disorder is a highly heterogeneous condition (20, 30). At least in part, this likely reflects the fact that several mechanisms of inheritance are involved in propagating the condition. Aside from complex interactions among the multitudes of single nucleotide polymorphisms incorporated into genetic networks, also known as genetic epistasis, structural genomic variations such as copy number variants and epigenetic variation all seem to play a role in the transmission of bipolar illness (20, 30, 31). Large-scale GWAS have scanned hundreds of candidate genes with variable results. Overall, GWAS have either failed to identify genes responsible for bipolar disorder, due to relatively small individual contributions, inadequate sample size, or disease heterogeneity (21, 30), or they have identified genes involved in “housekeeping” functions, such as translation, transcription, energy conversion, and metabolism (32). Genes involved in more brain-specific functions, including transmission, cell differentiation, cytoskeleton formation, and stress response have also been implicated (31). From these many studies, *CACNA1C*, a gene that codes for the alpha subunit of the L-type voltage-gated Ca^++^ channel, has been the most often replicated finding [Large-scale genome-wide association analysis of bipolar disorder identifies a new susceptibility locus near ODZ4 (20, 33, 34)]. Malfunction of *CACNA1C* has been associated with cognitive and attentional problems, both of which play a prominent role in bipolar psychopathology (35, 36). A handful of other genes, including *ODZ4*, coding for cell surface proteins involved in signaling and neuronal path finding, *NCAN*, a brain-expressed extracellular matrix glycoprotein (rodents with altered *NCAN* gene function show manic-like behaviors) and *ANK3*, a gene involved in localization of sodium channels, have had replicated GWAS support (20, 33, 34, 37, 38). Using a different approach, some studies have focused on gene networks and protein–protein interactions (39). For example, one study identified a set of disease markers for bipolar disorder that includes *SEC24C* (involved in vesicular transportation from endoplasmic reticulum to Golgi apparatus) and *MUSK* (which encodes proteins responsible for receptor assembly in the neuromuscular junction) (31). Several large studies have also implicated polymorphisms of clock genes in the etiology of bipolar disorder (40). Given the prominence of circadian dysregulation in cyclical mood disorders, the involvement of these genes in the condition seems plausible.

With the caveat that candidate gene approaches have been fast to identify risk genes for psychiatric disorders, but strikingly slow to replicate these findings when they occur, it is nonetheless of some relevance to review the most consistent findings from this approach. Candidate gene studies have identified a number of genes, such as catechol-*O*-methyltransferase (*COMT*), brain-derived neurotrophic factor (*BDNF*), neuregulin-1 (*NRG-1*), and disrupted in schizophrenia (*DISC-1*) that appear to be shared risk factors for schizophrenia, bipolar disorder, and MDD (41). In addition to *COMT*, bipolar disorder has been associated with polymorphisms in a number of other genes coding for monoamine receptors, transporters, and synthetic and catabolic enzymes, including monoamine oxidase (*MAOA*), dopamine transporter (*DAT*), serotonin transporter (*5HTT*), tryptophan hydroxylase (*TPH2*), and the *D2*, *D4*, *5HT4*, and *5HT2A* receptors (42–45). A polymorphism of the *5HTT* promoter has been linked with antidepressant-associated mania, lithium prophylactic efficacy, age of onset, and suicidality in bipolar illness (46–49).

A number of studies have found that the 66 Val/Met polymorphism of the *BDNF* gene, which has been associated with the regulation of neural resilience, plasticity, and proliferation, may be a risk gene for bipolar illness. Some of these studies have found a relationship between the BDNF polymorphism and brain morphology but not the disease state itself, whereas others have associated it with bipolar etiology only through an interaction with stressful life events (50–52). Furthermore, the BDNF polymorphism has been linked with disease severity, early adolescent onset, a propensity toward rapid cycling, and greater cognitive and executive function deficits in bipolar disorder (53–56).

Genes regulating glycogen synthase kinase-3 (GSK-3), a “pro-apoptotic” (programed cell death) peptide and a functional “opponent” of proteins involved in neuronal plasticity development, differentiation, and cytoskeletal assembly, have also been implicated in bipolar etiology. Researchers have reported an association between a *GSK-3* polymorphism and psychotic symptoms, the regulation of gene expression, lithium responsiveness, and alterations in white-matter microstructure in the context of bipolar illness (57, 58). Additional studies have reported linkages between genes regulating glutamate transmission (GRIN1, GRIN2A, GRIN2B, GRM3, and GRM4), the stress response (ND4, NDUFV2, XBP1, and MTHFR), inflammation (PDE4B, IL1B, IL6, and TNF), apoptosis (BCL2A1 and EMP1), and oligodendrocyte-mediated myelination of white-matter tracts (eIF2B) in bipolar disorder (42, 59–62).

Epigenetic changes reflecting an alteration of gene expression influenced by life events may play a significant role in different phases of bipolar illness (63). Indeed, studies have established a difference in the pattern of gene expression between the depressed state vs. euthymia or mania (64, 65). Furthermore, repeated manic episodes can cause oxidative damage to DNA, interfering with future DNA methylation, hence limiting the possibility of turning certain genes off (66). For example, hypomethylation of the *COMT* gene has been associated with both bipolar disorder and schizophrenia (67).

In summary, genetic studies of bipolar disorder have encountered numerous obstacles, in large part resulting from the need to bridge phenotypic and etiological heterogeneities. Evidence points to a complex polygenetic pattern of inheritance, involving a large number of genes with small to moderate individual effects, modified by epistasis, epigenetic modifications, and interactions with the environment. Findings have been inconsistent, but when positive have most often identified the “housekeeping” genes involved in cellular metabolic activities, ion exchange, synaptic development and differentiation, as well as genes regulating myelination, neurotransmission, neuronal plasticity, resilience, and apoptosis. It is conceivable that genetic influences may be reflected in an endophenotype (“hidden phenotype”) of bipolar disorder characterized by abnormal circadian and hormonal rhythms, responses to medications, and specific gray- and white-matter changes (42, 68–70).

## Studies of At-Risk Cohorts

Due to the progressive nature of bipolar disorder and the substantial morbidity and mortality, which accompanies this condition, it would be important to identify its presence as early in the disease course as possible. The last decade has seen a burgeoning research effort aimed at identifying genetic factors, phenotypical manifestations, biomarkers, and a pattern of imaging alterations, which would herald the onset of bipolar illness. Very sophisticated studies, including microsatellite and high-density SNP genotypes, combined with the whole genome sequence data of a large Old Order Amish pedigree sample, failed to identify a particular set of gene loci, which would identify at-risk individuals (71). Using a different approach, researchers reported an association between a bipolar polygenic risk score, derived from a large genome-wide meta-analysis of an MDD population, with several clinical features including early disease onset, severity, suicide attempts, recurrent and atypical depression, subclinical mania, and psychosis. However, it is important to note that the maximal variance in these traits attributable to this polygenic score was approximately in the 1% range (72). Although slight in its explanatory power, this polygenic analysis did confirm the findings of phenomenological literature focused on differentiating between bipolar disorder and MDD.

Attempts to predict bipolar disorder based on phenomenological criteria have met with variable success. One of these studies noted a predictive value for Childhood Bipolar Questionnaire items reflecting changes in Sleep/Arousal, Harm to Self and Others, Territorial Aggression, Anxiety, Self-esteem, Psychosis/Parasomnia/Sweet Cravings/Obsessions, and Fear of Harm (FOH). Children with FOH, compared to the ones without this risk trait had elevated indices of depression and mania, possibly reflecting a more severe future illness course (73). Another group validated ultra-high-risk criteria in a group of help-seeking adolescents (74). Utilizing bipolar-at-risk (BAR) criteria at baseline, which include items reflecting genetic risk (first degree relative suffering from bipolar disorder), depressive, cyclothymic, and sub-threshold mania features, investigators prospectively, over a 12-month period, predicted first episodes of mania/hypomania (74). While these are encouraging reports, two recent large meta-analyses concluded that it still not possible to accurately predict the development of bipolar disorder, based on the early phenomenology (75, 76).

On the other hand, emerging evidence suggests that brain imaging my hold predictive promise. For example, a radiological investigation comparing adolescents with high genetic risk for bipolar disorder and schizophrenia with matched controls, found evidence of significant reduction in coupling in both frontal–striatal and frontal–parietal networks, as well as lower recruitment of DLPFC during a sustained attention task (77). Furthermore, a reduction in the volume of orbitofrontal cortex, an area that plays a pivotal role in emotion regulation, was noted in healthy siblings of bipolar patients compared with healthy controls, suggesting its association with the heritability of this condition. Conversely, the same study discovered that a greater size of DLPFC may reflect resilience from bipolar illness, as it differentiated healthy from affected siblings (78). Finally, a promising line of research proposes a multifactorial approach, by developing a predictive algorithm based on familial/genetic factors, environmental adversity, early behavioral phenotype, biological markers [inflammatory cytokines, BDNF, markers of oxidative stress, and hypothalamic–pituitary–adrenal (HPA) disturbance], and imaging data (79–82).

## Neuroimaging in Patients with Bipolar Disorder

Neuroimaging studies of bipolar disorder are frequently characterized by equivocal findings and, in some instances, a failure to replicate previous results. Furthermore, multiple factors confound any attempt to integrate neuroimaging findings into a single theoretical paradigm. Chief among these is the fact that many studies fail to unequivocally define the mood state of patients at the time of scanning or fail to provide information on subjects’ age or medication status, all of which can impact brain activity. Differentiating the effect of medication on the underlying pathophysiological processes in bipolar disorder imaging studies is a daunting task. The majority of subjects in the imaging studies are medicated, quite often with more than one class of medication. In an ideal scenario, one would conduct a comparison between medicated and unmedicated bipolar patients and healthy control subjects. Such a design would require that medications be gradually reduced and discontinued prior to randomization. Ethical and clinical concerns would prohibit researchers from discontinuing medications in stable bipolar patients with a history of severe symptomatology, prominent suicidality, difficult-to-treat psychosis, highly recurrent disease, or rapid and polyphasic cycling. If these patients were to be excluded, it would introduce a selection bias, since the studies would represent only patients with a milder form of disease who could better tolerate medication discontinuation (83).

Importantly, two recent reviews addressing the impact of medication on imaging outcomes jointly indicate that medications have a fairly discrete impact in functional and diffusion tensor imaging studies. The impact of medication in functional imaging is difficult to discern, since majority of the study subjects are medicated, often with a combination of medicines. When there is a measurable medication effect on neural function in bipolar disorder, it is predominantly ameliorative or “normalizing” (83, 84). Findings of structural studies suggest an increase in volume of the brain areas involved in mood regulation, associated with lithium use, and mostly inconclusive effects of antipsychotics and anticonvulsants (84). Medication effects were more readily discernable in the longitudinal studies aimed at evaluating the medication effect on blood oxygen level-dependent (BOLD) signals (84). Most bipolar studies have been conducted in patients in euthymic or depressive states, given the difficulty of imaging floridly manic subjects, including the fact that imaging studies require minimal head motion. Furthermore, since research indicates that bipolar disorder is a progressive illness, possibly characterized by disparate pathophysiologic substrates in different phases of illness, one can make a case for stratifying the sample based base on the stage of the illness (85, 86).

However, despite these obstacles, there are some consistent findings regarding the impact of bipolar disorder on brain function and structure. Global structural brain changes and alterations in ventricle size have been a frequent finding, although not without exceptions (87). Magnetic resonance imaging (MRI) studies indicate that patients suffering multiple bipolar episodes had larger lateral ventricles relative to patients who only experienced a single episode or healthy controls (11).

## Changes in Ventricular Size and Cerebral Gray Matter Volume

Strakowski et al. utilized MRI to compare cerebral ventricle volumes in healthy controls vs. patients suffering their first bipolar episode or those who had experienced multiple episodes (11). Lateral ventricles were significantly larger in patients with multiple episodes than in the first episode or healthy subjects. In particular, increased volume of the lateral ventricles directly correlated with the number of manic episodes the patients had suffered. These findings have been supported by a different group of researchers who also noted an association between ventricular volume and number of previous affective episodes. Taken together, these studies indicate that bipolar illness may be progressive and deleterious, contributing to brain tissue deterioration in the course of recurrent episodes (8).

## Physiological Function of the Brain Networks Involved in the Pathophysiology of Bipolar Disorder

The clinical symptoms of bipolar disorder do not appear to be correlated to changes in the function or structure of specific brain areas. Rather, bipolar symptoms manifesting as emotional, cognitive, behavioral, autonomic, neuroendocrine, immune, and circadian disturbances better correspond to the dysfunction of interconnected brain networks (88–90). One perspective emphasizes a critical role of two interrelated prefrontal–limbic networks in the pathophysiology of bipolar illness. The first of these networks, commonly referred to as the Automatic/Internal emotional regulatory network, consists of an iterative loop, which includes the ventromedial prefrontal cortex (PFC), subgenual anterior cingulate cortex (ACC), nucleus accumbens, globus pallidus, and thalamus (this network has a significant overlap with the Salience network described by other authors). This network modulates amygdala responses to endogenously generated feeling states, such as melancholic feelings induced by memories of past losses.

**Figure 2:**
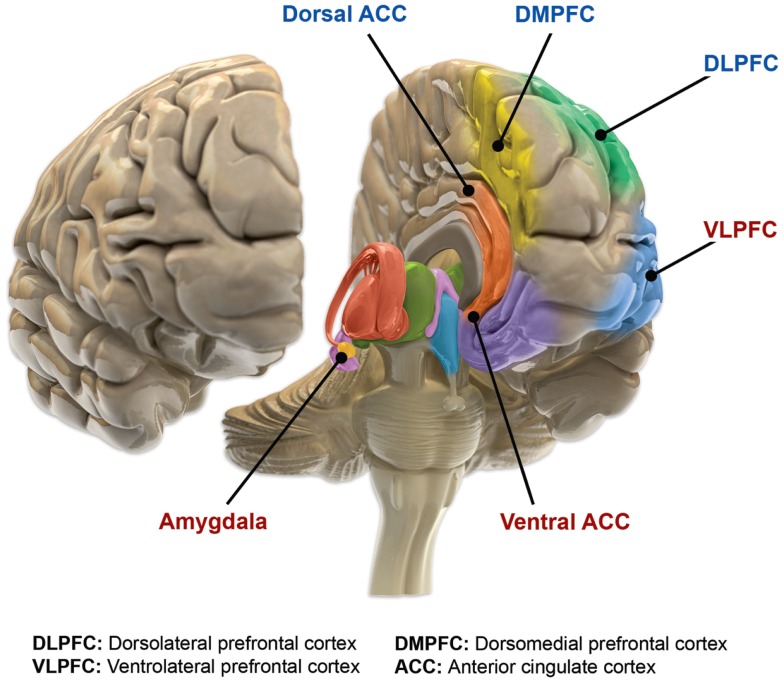
**Functional brain changes in bipolar disorder**. Based on Langan and McDonald (91). Illustration courtesy of: Roland Tuley, Fire and Rain. Imaging studies of euthymic bipolar patients provide evidence of compromised cognitive control, combined with increased responsiveness of limbic and para-limbic brain regions involved in emotional regulation. Brain areas associated with cognitive control, which manifest reduced responsiveness, are labeled blue (dorsal ACC, DMPFC, and DLPFC). By contrast, limbic and para-limbic brain areas involved in emotional regulation, associated with greater responsiveness, are labeled in red (amygdala, VLPFC, and ventral ACC).

The second of these networks, commonly referred to as the Volitional/External regulatory network, involves the ventrolateral PFC, mid- and dorsal-cingulate cortex, ventromedial striatum, globus pallidus, and thalamus (90). The dorsolateral PFC, with its connections to the ventrolateral PFC, is commonly described as the origination point of the volitional/cognitive regulatory arc (largely corresponding with Executive control network in other publications) (90). In turn, the ventrolateral PFC network modulates externally induced emotional states, assists with voluntary (cognitive) emotional regulation, and suppresses maladaptive affect. These two networks have shared components and collaboratively regulate amygdala responses in complex emotional circumstances (90). Components of this complex prefrontal–ACC–pallido-striatal–thalamic–amygdala network have altered function and structure in individuals suffering from bipolar disorder when compared with healthy populations (Figure 2). Specific changes in these structures will be reviewed in the following sections.

Another study compared within – and between – network connectivity in bipolar and schizophrenic patients vs. a healthy control sample (92). In addition to the Salience Network and Executive control network, the default mode network (DMN) has received a lot of attention in mood disorders, ADHD, and schizophrenia research. The DMN is composed of interconnected midline structures, including the sgACC, ventromedial PFC (vmPFC), dorsomedial PFC, precuneus/PCC complex, and mesotemporal structures. Some of the better studied functions of DMN include, self-reflection, processing social information, creative work, future planning, reminiscing, and conjuring of autobiographical memories. Calhoun et al. have noted impaired interconnectedness of the anterior DMN areas such as ventral ACC and vmPFC, with other DMN components in bipolar subjects, compared with schizophrenic patients and healthy controls. Imaging was conducted during an auditory selection task. Additionally, these investigators described altered functional connectivity related to bipolar disorder in cognitive/executive prefrontal and parietal areas (92). Albeit using different methodology, another group discovered aberrant resting state functional connectivity within the cingulo-opercular network and between, cingulo-opercular and cerebellar networks, and cerebellar and salience networks in bipolar patients compared to controls. Moreover, the latter two abnormal network connectivities correlated with disorganization symptoms in bipolar patients (93). The cingulo-opercular network is believed to play a role in the initiation and maintenance of task performance, as well as signaling the need for a change in cognitive strategy (93).

The most recent review of eight resting state functional network fMRI studies in bipolar patients reconfirms the findings of the above mentioned studies. The largest difference between bipolar patients and control groups were seen in the connectivity between ACC and mPFC, and the limbic structures. Furthermore, findings of aberrant intra-network homogeneity involving the DMN of bipolar patients, was also reproduced (94). While research into functional network connectivity has a potential to offer a better understanding of the neural origins of the complex cognitive, emotional, and physical symptoms of bipolar disorder, it requires consensus about the composition of functional networks, better control of confounding factors and more consistent, methodologically sound replications.

## Prefrontal Cortical Abnormalities in Bipolar Disorder

Prefrontal cortical abnormalities are a common finding in bipolar disorder. Imaging studies have reported functional and structural changes in the vmPFC of adolescents and young adult bipolar patients relative to healthy controls (95, 96). Dysfunction of vmPFC activity may be common to mood disorders and independent of mood state because it has been described in both unipolar (97, 98) and bipolar depression as well as in the context of elevated mood (95, 99). The vmPFC has rich reciprocal connections with limbic formations and the hypothalamus. Together with the ACC and amygdala, the vmPFC may belong to an integrative network involved in processing emotionally relevant information, which coordinates autonomic and endocrine responses and influences behavior (100). Aberrant vmPFC activity in the context of bipolar illness may therefore be reflected in compromised ability to adapt to changes in emotional and social circumstances. Manic patients tend to be excessively preoccupied by hedonic interests, whereas depressed individuals demonstrate impaired emotional and endocrine homeostasis. Furthermore, endocrine disturbances are also a common feature of elevated mood states (101). The vmPFC is also a source of feedback regulation to monoaminergic brainstem nuclei, so its malfunction may be reflected in altered neurotransmission (102).

The ventrolateral PFC is also often referred to as the lateral orbital PFC. This frontal area appears to have a role in the “top-down” and volitional regulation of affect, whereby it acts to suppress maladaptive emotional responses (102). Its activity has been reported to be both reduced and elevated in the depressive state (103, 104) but appears to be predominantly decreased in bipolar mania (90, 105, 106). Disinhibited and socially inappropriate behaviors, commonly present in mania, may be attributable to impaired ventrolateral PFC function (105). Most structural studies in bipolar adolescents have found a progressive reduction in the volume of this formation, whereas adult studies have provided equivocal findings (9, 87, 107). Some of the research indicates a combined effect of age and duration of illness on deterioration of volume in this brain region (9, 87, 107).

Decreased activity in the dorsolateral PFC in bipolar disorder may be associated with compromised working memory, impaired ability to sustain attention, and compromised executive function (103, 108). The dorsolateral PFC, together with the dorsal ACC and parts of the parietal cortex, is considered a component of the executive–cognitive network, which is known to exercise a regulatory role over limbic formations (109). A decline in the thickness of the dorsolateral PFC has been associated with bipolar illness duration (110).

The ACC is located at the intersection of dorsal (predominantly cognitive) and ventral (mostly emotion-regulating) cerebral regions (90). Additionally, the ACC serves as a sort of “anatomical bridge” that connects prefrontal cortical areas with subcortical limbic regions (9). The subgenual (sgACC) (Brodmann area 25), and subcallosal [alternatively labeled as pregenual (pgACC) anterior cingulate cortices (scACC) (BA 24a and b)] are sometimes jointly referred to as the ventral ACC. Rostral (rACC) and dorsal (dACC) (BA 24c and 32) are often identified as either dorsal ACC or more recently as mid-cingulate cortex (MCC) (111–114). As one would expect, dorsal and mid-cingulate areas are more involved in cognitive processes, whereas ventral portions of the ACC participate in emotional regulation. The dorsal, cognitive division of the ACC may be involved in tracking crosstalk or conflict between brain areas. If conflict is detected dACC may engage lateral prefrontal cortical areas in order to establish control operations (111). Pregenual ACC (pgACC) and anterior MCC are recipients of integrated intero- and exteroceptive information from anterior insula, in addition to amygdala input (115, 116). These integrative structures promote homeostatic efforts by maintaining a dynamic subjective image of the state of the body and the surrounding environment (116). Autonomic projections from sgACC to the amygdala, PAG, and nucleus tractus solitarius (NTS) in the medulla enable this “limbic” portion of ACC to instantiate an adaptive response to negative emotional events (113). There is some indication that ACC activation may be increased in mania and decreased in bipolar depression. Moreover, ventral portions of the ACC may be overactive even in the euthymic state, while dorsal segments remain hypoactive (90).

As one might predict from its location, the ACC plays a key role in cognitive–emotional integration and ongoing monitoring of behavior. The subgenual ACC orchestrates behavioral adaptation following an assessment of the salience of emotional and motivational information. The subgenual ACC also modulates bodily sympathetic and neuroendocrine activity in accordance with external conditions (117). The ACC and insula are the two primary hubs of the Salience network, tasked with detecting relevant changes in the internal and external environment and generating an appropriate emotional response (118). Inappropriately modulated emotional responses to changes in the environment and motivational difficulties in bipolar disorder may be associated with altered ACC function and structure (119). Structural studies have noted significantly decreased volume in the subgenual ACC in bipolar patients (119). Some authors have speculated that early morphological abnormalities of the ACC may be markers of vulnerability for ensuing psychosis and emotional dysregulation (9).

The imaging literature is beset with inconsistent findings regarding hippocampal volume in bipolar disorder. Some studies have found enlargement, others have noted loss of volume, and others have reported no difference in hippocampal size in bipolar patients compared with controls (107, 120). There is some indication of an age-related increase in hippocampal volume in bipolar youths (121), and mood-stabilizing agents (e.g., lithium) have been reported to increase hippocampal volume (122, 123). Decreases of hippocampal volume in the adulthood of bipolar individuals may be driven by a polymorphism of genes regulating BDNF function and may be localized to certain hippocampal substructures (9, 107, 121).

As in other brain areas, structural changes of the amygdala may reflect the progression of bipolar illness. Most of the volumetric studies have reported that bipolar children and adolescents have a smaller amygdala volume, whereas adults have a larger volume, compared with matched controls (107, 124). Changes in amygdala volume in adulthood may reflect the progressive course of bipolar illness or may be a consequence of an ameliorative effect of medication (9, 107). Functional studies have, for the most part, found increased activity in limbic structures of bipolar patients in both the manic (a more consistent finding) and depressed state (detailed discussion will follow in this section). The amygdala is involved in the assessment and interpretation of emotion, particularly the emotional value of surprising or ambiguous stimuli. Clinical studies have provided evidence that patients with bipolar disorder often have disproportionate emotional responses to changes in circumstances and difficulty interpreting the emotional meaning of facial expressions (124). Because limbic structures have significant bidirectional connections with the hypothalamus and autonomic bed nucleus of the stria terminalis, one might speculate that limbic dysregulation may contribute to the often-noted autonomic and neuroendocrine dysregulation in bipolar patients (125).

Several subcortical structures appear to be affected by bipolar illness. Functional imaging studies have reported decreased activation of caudate, putamen, thalamus, and globus pallidus in bipolar patients performing a response inhibition task, and attenuated ventral striatum responses to happy faces across the mood states (90). A recent meta-analysis has provided conflicting data regarding basal ganglia and thalamic activation in bipolar illness (126). Volumetric imaging studies have provided evidence of decreased nucleus accumbens in bipolar individuals compared with matched healthy controls (87). Studies on morphological changes of the basal ganglia and thalamus in bipolar disorder are both sparse and contradictory (9, 87, 107). Although one of the studies discovered enlargement of the anterior putamen and head of the caudate in bipolar disorder, other researchers found no difference in volume (9, 87, 107).

Rare functional studies have described either attenuated cerebellar activity in bipolar disorder or no difference from healthy controls (126). Limited structural imaging studies have indicated midline cerebellar atrophy in bipolar subjects. Vermal size appears to be associated with the number of previous mood episodes (9, 87, 107, 120). Changes in cerebellar function and structure may be of particular clinical relevance in bipolar disorder because the cerebellar vermis has been linked to the production of automatic emotional responses, including empathy with facial expressions (125). Furthermore, cerebellar–thalamic–basal ganglia–cortical circuits have been implicated in reward-based learning (127), so their altered function may provide an explanation for the significant association between bipolar illness and substance use disorders.

Diffusion tensor imaging studies evaluating white-matter tract microstructure in bipolar disorder have found widespread abnormalities (128). Several studies have detected alterations in white-matter tracts connecting the subgenual ACC with the amygdala–hippocampal complex, frontal lobe–insula–hippocampus–amygdala–occipital lobe and frontal lobe–thalamus–cingulate gyrus in bipolar patients relative to healthy controls (128–131). Furthermore, altered white-matter connectivity between the dorsal/medial ACC and posterior cingulate cortex, as well as between the dorsolateral PFC and orbital PFC, has been detected in bipolar disorder patients compared with healthy subjects (132). Finally, disruption of white-matter fibers connecting both medial (automatic) and lateral (volitional) PFC networks with amygdala, striatum, and thalamus in bipolar patients relative to healthy subjects may reflect global deficits in prefrontal regulation of limbic areas (129). The anatomical locations of these white-matter abnormalities are consistent with clinically observed impulsivity, affective reactivity, and aberrant processing of emotional stimuli (128–131). White-matter changes appear to be asymmetrical and present in the earliest stages of bipolar illness, most likely indicating abnormal expression of myelin- and oligodendrocyte-related genes (128–131, 133, 134). Consistent with these observations, studies have established white-matter abnormalities in at-risk children and impaired frontal white-matter integrity in first-episode manic patients (90). In aggregate, white-matter studies suggest a developmental disturbance that precedes and possibly predisposes to mood dysregulation and eventual onset of bipolar episodes (90). Furthermore, white-matter changes may be state dependent, as one study reported ventromedial prefrontal–striatal, inferior fronto-occipital, and inferior and superior longitudinal fasciculi white-matter alterations in the bipolar depressed state, differentiating it from both remitted patients and healthy controls (135).

## State or Trait? – Changes in Brain Function and Structure in Bipolar Mood States

Neuroimaging studies have provided a more detailed, although still incomplete, understanding of the pathophysiological processes that underpin different mood states in bipolar disorder. An increase in amygdala activity is a frequently described feature of elevated mood in bipolar disorder (90). Although many studies using activation paradigms noted an increased amygdala response in mania, resting-state imaging did not detect increased amygdala activity compared with healthy controls (105, 106, 136). Furthermore, several (but not all) imaging studies reported elevated dorsal ACC activity in the context of bipolar elevated mood compared with depressed patients or healthy individuals (90, 136, 137). Several other limbic and paralimbic areas, including insula, hippocampus, putamen, and subgenual ACC, have been noted to have greater activity in manic subjects than in healthy controls (136, 137). Decreased ventrolateral PFC activity is another common feature of bipolar mania that differentiates it from depressed, euthymic state, and healthy controls (99, 105, 106, 137). Further extending these observations, a group of investigators has reported that a decrease in ventrolateral PFC activation correlated with the duration of the manic episode (105). Diminished activity of rostral PFC either in the resting state or in response to negative emotional stimuli was detected in mania compared with healthy controls (99, 136, 138). Moreover, resting-state hypoactivity of dorsolateral PFC has been associated with mania in comparison with healthy controls (106, 136). In summary, impaired prefrontal cortical function in elevated mood states may result in compromised regulation of limbic and paralimbic areas, manifesting as excessive emotional reactivity, irritability, impulsivity, difficulty conforming emotional responses to the social milieu, excessive indulgence of appetitive drives, and cognitive/attentional impairment.

Bipolar depression shares some activity patterns with elevated moods but also has some distinguishing features. Bipolar depressed patients have demonstrated a greater amygdala response to negative facial expressions than manic or healthy individuals (137). Several studies have noted elevated activity in other limbic and subcortical areas, including insula, ventral striatum, putamen, hypothalamus, and thalamus, in bipolar depression relative to healthy controls (106, 137, 139). In support of this observation, a magnetic resonance spectroscopic (MRS) study revealed elevation of glutamate/glutamine signal in the thalamus of bipolar depressed patients (140). Others have reported conflicting findings of diminished metabolism/blood flow in the insula, ventral striatum, and subgenual ACC of depressed bipolar patients (141). Most studies note diminished prefrontal cortical activity in dorsolateral PFC, ventrolateral PFC, and dorsomedial PFC in depressed bipolar patients compared with either euthymic patients or healthy controls (90, 106, 141, 142). Decreased dorsolateral PFC activity during a working memory task correlated with the severity of depression in bipolar patients, measured by a standardized scale (143). Both elevation and decrease of vmPFC activation have been detected in bipolar depression (138, 142). Interestingly, a recent MRI study comparing bipolar depressed with euthymic patients discovered decreased gray-matter volume of dorsomedial PFC and dorsolateral PFC (144). These morphological alterations completely mirror decreased function in dorsomedial PFC and dorsolateral PFC and provide strong support for the hypothetical impairment of neuroplasticity in bipolar disorder (144). In conclusion, imaging data suggest that compromised activity in prefrontal cortical areas may result in inadequate modulation of limbic/subcortical areas, especially in response to negative life events, contributing to maladaptive depressed mood and inadequate cognitive coping. Imaging data have so far provided evidence that clearly distinguishes depression from the other mood states in bipolar disorder.

Most of the studies examining neural function in the euthymic state have noted decreased function in ventrolateral PFC, dorsolateral PFC, and hyperactivation of striatal regions (caudate and putamen) (106). A couple of resting-state imaging studies have made some intriguing discoveries. One group noted significant hyperconnectivity between ventrolateral PFC and amygdala that is, to a lesser degree, also modulated by connectivity through the ACC (145). Aberrant connectivity of these components of the volitional/external cortico-limbic network may be a trait feature of bipolar disorder, possibly predisposing toward future mood instability in the face of stressful events. Moreover, a different group, also utilizing functional imaging in resting-state euthymic, older bipolar adults, discovered increased amygdala, parahippocampal, and anterior temporal cortical activity, combined with decreased dorsolateral PFC activity. Most of these findings are absent in the younger euthymic bipolar population, pointing to the progressive nature of bipolar disorder, whereby cortico-limbic dysfunction becomes consolidated over time into a trait-like pattern of activity (146).

## Imaging Differences between Bipolar and Unipolar Depression

Discriminating between bipolar and unipolar depressive episodes remains a clinical challenge. Recent imaging studies may indicate some important differences in the pathophysiology of these conditions. An fMRI study used images of happy, sad, and neutral facial expressions as a stimulus. Patients with unipolar depression manifested increased amygdala activation in response to negative facial expressions, whereas patients with bipolar depression demonstrated a greater amygdala response to positive facial expressions (147). Another study used fMRI to analyze whole-brain patterns of activation and also noted that viewing intensely happy faces generated an activity pattern that differentiated bipolar depression from MDD (148). Consistent with these observations, a different group of authors noted greater amygdala activation in response to angry expressions in MDD patients relative to a bipolar depressed group (149). Furthermore, activation of medial and orbitofrontal prefrontal regions in response to emotional stimuli contributed to the diagnosis of unipolar depression (147). This finding is very intriguing because both of these ventral PFC areas are components of a neural network involved in the “automatic”/internal regulation of emotion (90). Greater activation in dorsolateral and ventrolateral prefrontal areas in response to positive and negative emotional features contributed to a classification of the subject as having bipolar depression. Both of these lateral PFC structures play a critical role in volitional/external emotional regulation and have been shown to have an exaggerated responsiveness to emotional stimuli in the context of bipolar disorder (90). A computerized automatic algorithm utilizing the above-mentioned information was able to correctly categorize unipolar vs. bipolar depression with up to 90% accuracy (147).

Connectivity between other components of the ventrolateral and ventromedial prefrontal networks (prefrontal–cingulate–striatal–pallidal–thalamic–amygdala) may also differentiate unipolar and bipolar depression (90, 150). The ACC is at the crossroads between ventral (mostly emotional) and dorsal (predominantly cognitive) networks connecting prefrontal regulatory with subcortical integrative brain regions (90, 150). Bipolar and unipolar depressed patients had significantly decreased pgACC connectivity with dorsomedial thalamus, amygdala, and pallido-striatum compared with healthy controls. Compared with unipolar depression, bipolar depressed subjects had significantly decreased connectivity between pgACC and amygdala and dorsomedial thalamus (150) Moreover, a separate group of investigators reported a more intense activation of ventral striatal, thalamic, hippocampal, amygdala, caudate nucleus/putamen, vmPFC, ventrolateral PFC, and ACC in bipolar depressed individuals compared with MDD and a healthy control group, especially in response to mildly and intensely fearful and sad, and mildly happy expressions (104). In aggregate, these findings may reflect a greater degree of impairment in the prefrontal–cingulate–striatal–pallidal–thalamic–amygdala circuits in bipolar vs. unipolar depression. Although evidence substantiates impairment of both volitional and automatic prefrontal–limbic circuitry, the volitional ventrolateral PFC-mediated network seems to be more compromised in bipolar than unipolar depression, possibly reflecting compromised prefrontal regulation of the subcortical limbic areas, manifested as more prominent emotional lability and reactivity in this disease state.

In addition to functional differences, there are also structural differences between unipolar and bipolar depression. Compared with bipolar depressed subjects, those with MDD had fewer deep white-matter hyperintensities, reflecting a lesser degree of white-matter impairment. Additionally, bipolar depressed subjects had increased corpus callosum cross-sectional area and decreased hippocampus and basal ganglia relative to unipolar patients. Both disorders manifested a larger lateral ventricular volume and increased rates of subcortical gray-matter hyperintensities compared with healthy controls (151).

## Summary of Imaging Findings in Bipolar Disorder

Cumulative imaging evidence of functional, structural, and white-matter abnormalities implicates a compromised integrity of frontal–subcortical and prefrontal–limbic circuits in the pathophysiology of bipolar disorder. Additional involvement of frontal–basal ganglia–thalamic–cerebellar networks is likely. In summary, structural and functional changes support an organic basis for the emotional, cognitive, and neuroendocrine symptomatology of bipolar illness (89, 90, 119). Both regional gray-matter and white-matter changes appear to be present relatively early in disease development. Altered emotional homeostasis and cognitive difficulties stemming from these prodromal functional changes may compromise stress coping and social adaptation, hastening the onset of bipolar illness. In some instances, there is evidence of a cumulative effect of disease duration and the number of prior episodes of brain function and structure.

## Pathohistologic Findings Associated with Bipolar Disorder

Pathohistologic research has uncovered significant cell pathology associated with bipolar disorder. It appears that all three of the glial cell families may be affected, linking the pathogenesis of the condition to abnormalities in astroglia, oligodendroglia, and microglia (152–155). Postmortem studies of bipolar patients have noted a reduction in both glial cell numbers and density (156). Glial alterations have been reported in the subgenual ACC, dorsolateral PFC, orbitofrontal cortex, and the amygdala of unmedicated bipolar patients (157, 158). Interestingly, one study found evidence that treatment with lithium or valproate may mitigate some of the glial loss (157). Furthermore, a significant 29% reduction in oligodendroglia numerical density in the dorsolateral PFC white matter was detected in bipolar patients compared with controls (155). Evidence of diminished myelin staining in the dorsolateral PFC and reductions of S100B immune-positive oligodendrocytes in the hippocampus of bipolar subjects further extend these findings (159, 160). Indeed, convergent imaging, histologic and imaging evidence indicates that oligodendroglial deficits may be the key CNS cellular abnormality in bipolar disorder (60, 61, 161–163).

A postmortem study of suicidal bipolar, MDD, and schizophrenic patients provided intriguing insights into a possible role for microglia in the pathophysiology of these conditions. Unlike mood-disorder patients who committed suicide, subjects who had the same diagnosis but died of other causes showed no evidence of brain microgliosis. However, suicidal mood-disorder patients, including the bipolar group, had a substantial elevation in microglia density in the dorsolateral PFC, ACC, and mediodorsal thalamus when compared with both controls and mood-disorder patients who did not die by suicide (154). Given the established role of microglia in CNS inflammation, these findings raise the intriguing possibility that suicidality might literally be a consequence of the disease flare-up. Supporting a role for these microglia changes in disease pathology is the fact that a remarkable overlap exists between the sites of cellular pathology and the brain regions with altered structure and function in neuroimaging studies of bipolar illness (164).

In contrast to the evidence for a glial role in bipolar pathogenesis, the data supporting a role for a primary neuronal pathology in the condition are less convincing. With a few notable exceptions, neuronal changes in bipolar disorder are mostly morphological in character, possibly attributable to apoptosis and thinning of interneuronal neuropil (164), and are much less extensive than glial pathology. Nonetheless, one study reported a 16–22% decrease in neuronal density in the dorsolateral PFC of bipolar disorder patients compared with a control group (165). These large pyramidal cells are glutaminergic excitatory neurons (165). It bears reminding that dorsolateral PFC pyramidal neurons are the main target of thalamic projections and also provide regulatory feedback to the amygdala and ACC. These connections make it likely that neuronal dorsolateral PFC pathology may result in compromised attention, executive function, and top-down emotional regulation, all of which are prominent features of bipolar illness. Additionally, studies have detected a significant reduction in neuronal density in the hippocampus and a prominent decrease in neuronal size in the ACC of bipolar subjects relative to controls (166, 167).

Several studies have examined changes in monoaminergic nuclei that may affect mood regulation. Patients with bipolar disorder appear to have a higher number of noradrenergic neurons in the locus ceruleus as well as subtle structural deficits of serotonergic neurons in the dorsal raphe (164).

In summary, the available evidence does not provide much support for viewing bipolar disorder as a typical neurodegenerative disease. Unlike conventional neurodegenerative disorders, which are associated with prominent neuronal loss and prominent gliosis, glial loss seems to be the dominant cellular pathology in bipolar illness (164). In other words, if we have to use a label, bipolar disorder is much more a “gliopathic” rather than a neurodegenerative condition. Although the clinical correlates of the cellular pathology in bipolar disorder await better characterization, there is little doubt that documented pathohistological changes in key cortico-limbic areas and the white-matter tracts play an important role in the clinical manifestations of bipolar illness.

## Neuroendocrine and Autonomic Dysregulation in Bipolar Disorder

Alterations in HPA axis function in bipolar disorder have been well substantiated (168). Exaggerated release of corticotropin-releasing factor (CRF) contributes to greater adrenocorticotropic hormone (ACTH) secretion and a subsequent elevation of circulating glucocorticoids (i.e., cortisol) (168). These disturbances are most likely attributable to deficits in cortico-limbic regulation in bipolar disorder, with consequent amygdala over-activity, and a compromised hippocampal regulatory role (169). Moreover, glucocorticoid receptors appear to have diminished sensitivity in mood disorders, possibly due to elevation in inflammatory cytokines, thereby disrupting physiological feedback regulation on the HPA axis and immune system (170–172). Indeed, even euthymic bipolar patients exhibit a flattening of the cortisol curve (an ominous indicator of compromised overall health) compared with healthy controls. In patients unfortunate enough to have suffered multiple episodes, these abnormalities intensify, resulting in higher overall cortisol levels in addition to aberrant reactivity, and even greater flattening of their cortisol curves, compared with patients who have experienced only a few episodes (173). Highlighting the relevance of these neuroendocrine abnormalities, a recent study has associated elevated evening cortisol levels in bipolar individuals with a history of suicidal behavior (174).

In addition to HPA dysregulation, bipolar disorder may be associated with excessive sympathetic nervous system (SNS) activity. For example, extra-neuronal norepinephrine was reported to be elevated in a group of bipolar patients relative to healthy controls (175). Autonomic dysregulation, more generally reflected by decreased parasympathetic activity and elevated sympathetic activity, may be a trait marker for bipolar disorder, as indicated by a report of markedly lower heart rate variability in euthymic bipolar patients than in healthy controls (176). The constellation of SNS overactivity, parasympathetic withdrawal, glucocorticoid receptor insufficiency, and elevated inflammatory signaling may help account, at least in part, for the increased risk of metabolic syndrome, endocrine disorders, and vascular disease seen in bipolar patients (168, 177). Highlighting the relevance of this pattern of neuroendocrine, autonomic and immune changes is the fact that vascular disease has recently been identified as the leading cause of excess death in bipolar disorder (178).

In addition to affecting autonomic and immune function, elevated glucocorticoids have been associated with suppression of thyroid-stimulating hormone secretion and compromised enzymatic conversion of relatively inactive thyroxine to active triiodothyronine (172). An ensuing low-grade thyroid dysfunction has been associated with bipolar disorder and most likely influences both the clinical presentation and the treatment response (179, 180).

## Circadian Dysfunction in Bipolar Disorder

Multiple lines of evidence indicate a relationship between bipolar disorders and circadian dysregulation. Circadian disturbances are not likely to be an epiphenomenon of bipolar illness given that they are present during mania, depression, in euthymic state, and in healthy relatives of bipolar patients (181, 182). Actigraphic evidence and polysomnography studies have detected higher density of REM sleep, greater variability in sleep patterns, longer sleep latency and duration, lower sleep efficiency, greater number of arousals, fragmented sleep, and reduced daily activity, both in actively ill and remitted bipolar patients, relative to healthy controls (181–184). One study found delayed sleep phase in 62% of bipolar depressed, 30% of MDD, and 10% of control subjects (185). Other authors have pointed out that bipolar sufferers have inherent instability and blunting of biological rhythms, rendering them intolerant of shift work (186). Diminished sleep efficiency and rhythm robustness in bipolar disorder patients have been recently linked with abnormal dorsolateral prefrontal cortical response and impaired performance on a working memory task compared with healthy controls (187). Furthermore, an irregular and delayed sleep wake cycle has been associated with the lifetime emergence of hypomanic symptoms in a non-clinical adult sample (188).

An evening preference, in morningness–eveningness typology, has been linked with bipolar disorder. Biological chronotype tends to be strongly associated with biomarkers such as salivary melatonin, morning cortisol, catecholamine secretion, and changes in body temperature. Moreover, eveningness has a significant correlation with important clinical manifestations of bipolar illness, including intensity of depression, rapid mood swings, anxiety, substance abuse, a greater sensitivity to sleep reduction, daytime lethargy, and reduction in melatonin levels (181, 182, 186).

Altered endocrine and neurotransmitter diurnal rhythms in bipolar disorder have also been described. In physiological circumstances circulating melatonin increases approximately 2–3 h before sleep, remains elevated during nighttime sleep and rapidly decreases in the morning before awakening. Circulating cortisol is typically contra-correlated with melatonin. While high morning cortisol levels assist with the wakening effort, low evening cortisol supports preparation for sleep. Although there are contradictory findings, bipolar patients may have a hypersensitive melatonin response to light. In response to light exposure, both euthymic and actively affected bipolar patients manifest two-fold greater reduction of nocturnal plasma melatonin concentrations compared with the healthy controls (181, 182, 186). Furthermore, many bipolar subjects have substantially delayed and reduced melatonin secretion compared to MDD patients (189). Disturbances in diurnal glucocorticoid regulation were already discussed in the previous section, we will just add that bipolar patients have higher awakening and evening cortisol than control groups. Even offspring of bipolar parents have higher afternoon salivary cortisol compared to healthy controls (181, 182).

The secretion of several neurotransmitters is subject to circadian regulation and appears to be altered in bipolar disorders. There are rich bidirectional connections between serotonergic nuclei raphe and the main circadian pacemaker, the hypothalamic suprachiasmatic nucleus (SCN). Both serotonin and melatonin levels peak at night. The pace of conversion of serotonin to melatonin is regulated by SCN. Moreover, serotonin synthesis is subject to significant diurnal and seasonal rhythmic fluctuations. Serotonin, in turn, has been found to influence transcription of the CLOCK genes in a preclinical model (181, 182). Ventral tegmental area dopaminergic neurons have been implicated in regulation of REM sleep and adaptation to light, while norepinephrine provides a regulatory influence on melatonin synthesis (181, 182). The relationship between bipolar disorder and the role of monoamines in the disturbance of circadian regulation requires further exploration.

In contrast to large scale GWAS which have not established an association between CLOCK genes and bipolar disorder, smaller linkage studies, while lacking adequate replication, have noted an association between several circadian genes, including TIMELESS, ARNTL1, PER3, NR1D1, CLOCK, and GSK-3 beta, and the bipolar illness (181, 182, 186).

Finally therapeutic interventions, focused on restoring proper circadian rhythmicity, such as interpersonal and social rhythms therapy (IPSRT) and phototherapy have received preliminary empirical support. The use of phototherapy in bipolar disorder is beset with controversy, as it has been reported to precipitate serious adverse responses, such as mood instability, suicidality, and mania (186). Controlled studies have indicated efficacy of the IPSRT approach in extending time to recurrence of bipolar episodes and improvement in occupational functioning. Conversely, alteration in treatment was associated with an increased risk of recurrence (186). However, rigorous randomized controlled replication of these findings will be necessary before they become a part of routine clinical practice.

## Immune Disturbances in Bipolar Disorder

Several limbic and paralimbic areas implicated in the pathophysiology of bipolar illness, including amygdala, insula, and ACC, have an important role in the regulation of autonomic and immune function (102, 190–192). Although direct data are not available linking disturbances in these limbic/paralimbic areas to inflammation in bipolar disorder, it tempting to speculate that their aberrant activity may have a causal role in the ensuing immune dysregulation that has been repeatedly observed in patients with bipolar illness. Several studies and two recent meta-analyses have reported elevated levels of peripheral inflammatory cytokines in bipolar depressed and manic patients compared with healthy controls (193–199). Both meta-analyses indicated higher levels of tumor necrosis factor (TNF)-alpha and IL-4 in bipolar subjects relative to healthy subjects (196, 197). Elevation of IL-4 was noted only in the studies that did not utilize mitogen stimulation, while stimulated studies demonstrated no difference in IL-4 levels between bipolar and healthy subjects (196). IL-4 induces transformation of naïve helper T-cells into Th2 cells and reduces production of Th1 cells and macrophages. As such, IL-4 is a key “switch” regulating the balance between cellular and antibody-based immunity. One might speculate that IL-4 elevation in bipolar disorder may be of compensatory nature, to buffer against the increase of proinflammatory cytokines seen in the condition. More rigorous controlled studies, accounting for medication use and mood state effects need to be done before we can arrive at a better understanding regarding the role of IL-4 in bipolar disorder.

Separate studies have found that both bipolar manic and depressed patients have higher levels of TNF-alpha and IL-6 compared with matched controls (193, 195, 199). Moreover, research has established that other inflammatory markers such as high-sensitivity C-reactive protein and chemokines tend to be elevated in the course of bipolar episodes (200, 201). In addition to the elevation of proinflammatory IL-6, common to both bipolar mood states, bipolar depression relative to bipolar mania is characterized by an altered balance between IL-6 and the anti-inflammatory IL-10 (193). Based on published values, the IL-6/IL-10 ratio is 1:18 in mania and 2:44 in bipolar depression (193). Overall, the data suggest that successful treatment leading to a euthymic state may reverse inflammation and normalize peripheral levels of inflammatory mediators (195, 196, 202). Inflammatory cytokines are a known cause of diminished sensitivity of glucocorticoid and insulin receptors (172). Combined with autonomic disturbance, increased platelet/endothelial aggregation and unhealthful lifestyle, elevated inflammation may contribute to substantially increased risk of respiratory and gastrointestinal disorders, cerebrovascular and cardiovascular disease, and migraines in the bipolar population (79, 168, 203). The cumulative impact of impaired HPA regulation combined with compromised glucocorticoid and insulin receptor activity, aggravated by inflammatory cytokines, might explain the high rate of metabolic syndrome, diabetes, dyslipidemia, and osteoporosis in the bipolar population (88, 204). Furthermore, increased peripheral inflammation has been associated with numerous symptoms of mood disorders, such as malaise, fatigue, anhedonia, impairment of concentration, anxiety, irritability, social disconnection, hopelessness, suicidal ideation, bodily aches, and disturbance in sleep and appetite (204–208).

Peripheral inflammatory signals can gain access to the CNS through several pathways, as follows: (1) several brain areas are not “covered” by the blood–brain barrier (BBB); (2) afferent vagal fibers may convey the peripheral inflammatory signals to their nuclei, including nucleus tractus solitarii; (3) BBB cells have the ability to import cytokines via active transport; (4) peripheral immune cells such as macrophages, T-lymphocytes, and monocytes may gain access to the CNS and release the inflammatory mediators; and (5) BBB cells (endothelial cells and pericytes) can be induced to release inflammatory signals (209). Elevation of cerebrospinal fluid (CSF) inflammatory cytokines (IL-1beta) has been substantiated in bipolar patients, especially if they have experienced recent manic episodes, compared with healthy volunteers (171). Imaging studies have reported peripheral inflammation-related changes in the activity of several limbic and paralimbic areas, including subgenual ACC, amygdala, medial PFC, and basal ganglia/ventral striatum/nucleus accumbens (209–211). All of these limbic/paralimbic areas involved in the regulation of mood and stress response have also been implicated in the pathophysiology of bipolar disorder. These referenced studies have clear limitations, since an elevation of peripheral inflammatory cytokines was elicited by the injections of typhoid vaccine or endotoxin (210, 211), and therefore cannot be readily transposed to the processes that take place in the context of mood disorders. Nonetheless, it is intriguing that elevation of inflammatory cytokines in the CNS has been associated with suppressed synthesis of neurotrophic factors (especially BDNF) and compromised monoaminergic transmission (204), both of which have been reported in bipolar disorder.

**Figure 3:**
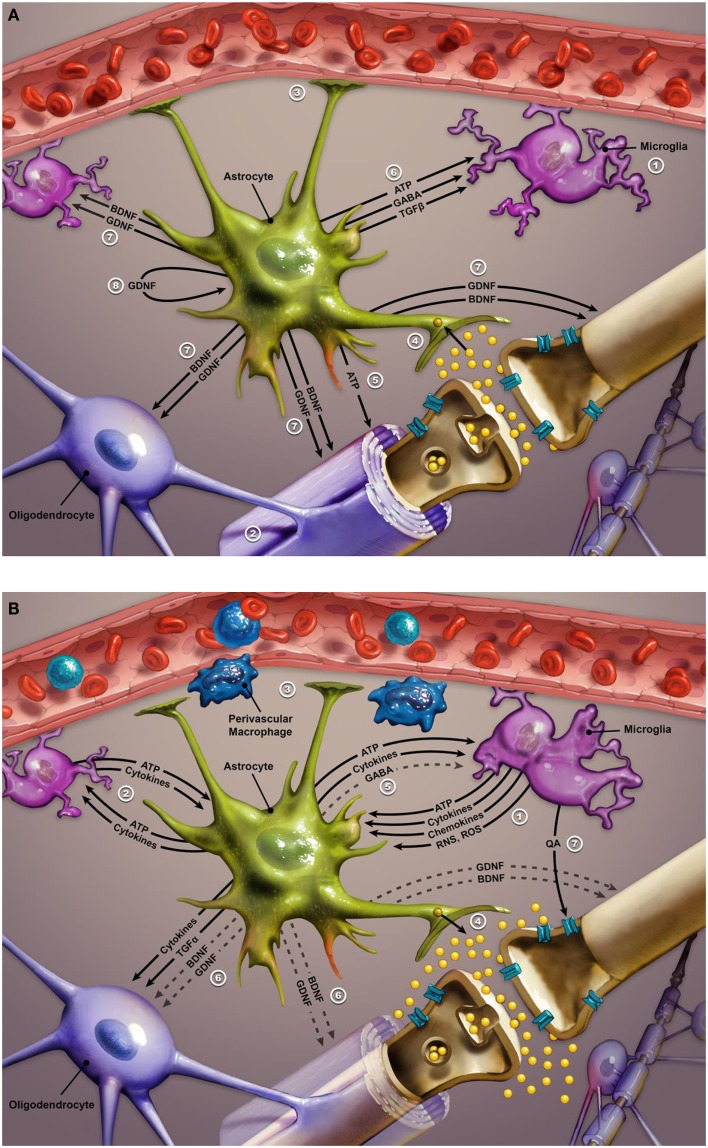
**(A)** Glial–neuron interactions: normal conditions. Glial–neuron interactions under non-inflammatory conditions. This image illustrates the relationship between glial cells and a “typical” glutamate neuron. Numbers in legend refer to corresponding numbers on image. Glial cell functioning is critical to sustaining and optimizing neuronal functioning in the CNS. The three types of glial cells are microglia, oligodendrocytes, and astroglia. Microglia act as ambassadors of the immune system (1), monitoring for derivatives of peripheral inflammatory signals. Oligodendrocytes optimize neuronal signaling by myelination of neurons (2). Astrocytes serve a number of functions including: maintenance of the blood–brain barrier and facilitation of neurovascular coupling (3); protection of the neuronal synapse (4) by removing excess ions to keep firing rate steady and removing excess glutamate before it can diffuse out of the synapse to bind to extrasynaptic NMDA receptors (which are implicated in neurotoxicity); (5) release of ATP to reduce neuronal glutamate release; (6) calming and stabilizing microglia via release of ATP, GABA, and TGFβ; providing trophic support via BDNF and GDNF to neurons, microglia, and oligodendrocytes (7). GDNF released by the astrocyte also supports the functioning of astrocytes themselves via an autocrine signaling pathway (8). Abbreviations: NMDA, *N*-methyl-d-aspartate; GABA, gamma aminobutyric acid; TGFβ, transforming growth factor beta; BDNF, brain-derived neurotrophic factor; GDNF, glial cell-derived neurotrophic factor; ATP, adenosine triphosphate. **(B)** Response to dysregulated peripheral inflammatory signals. Response to peripheral inflammatory signals. Microglia detect derivatives of the peripheral inflammatory signals such as ones conveyed by perivascular macrophages, and propagate/transduce this signal to the central nervous system through release of ATP, cytokines, chemokines, RNS, and ROS (1). The inflammatory mediators released by microglia initiate a positive-feedback loop in which astrocytes also begin to release ATP and cytokines, which triggers further inflammatory cytokine release from microglia, thus perpetuating the inflammatory cycle (2). Increased levels of ATP and inflammatory mediators lead to a cascade of events that result in destabilization and impairment of the normal functioning of both glial and neurons. Astrocytes become unable to maintain the integrity of the blood–brain barrier and optimal neurovascular coupling (3). Instead of removing excess glutamate from excitatory synapses, activated astrocytes release additional quantities of this neurotransmitter, producing an excess of glutamate that may impair synaptic communication (4) and lead to excitotoxicity via stimulation of extrasynaptic NMDA receptors. Activated astrocytes decrease release (indicated by dashed lines) of GABA (5) which results in destabilization of microglia such that they become amoeboid in shape and able to move throughout the brain while continuing to release inflammatory cytokines and ATP. Activated astroglia also reduce the release of neurotropic factors, such as BDNF and GDNF (6). The decline in BDNF and GDNF further perpetuates microglia activation, precipitating impairment in oligodendrocyte function and demyelination, as well as neuronal apoptosis. Activated microglia exhibit increased activity of the enzyme indoleamine 2,3-dioxygenase, which eventually converts tryptophan into quinolinic acid (QA). Increased metabolism of tryptophan to quinolinic acid may interfere with serotonin signaling due to depletion of tryptophan, while released quinolinic acid contributes to neurotoxicity via stimulation of extrasynaptic NMDA receptors (7). Abbreviations: RNS, reactive nitrogen species; ROS, reactive oxygen species; ATP, adenosine triphosphate; BDNF, brain-derived neurotrophic factor; GDNF, glial cell-derived neurotrophic factor; GABA, gamma aminobutyric acid; TGFα, transforming growth factor alpha.

Inflammatory cytokines activate microglia in the brain, causing their phenotypical transformation. Active microglia amplify inflammatory signals by releasing reactive oxygen species, reactive nitrogen species, cytokines, and chemokines (see Figure 3). This chemical cocktail of oxidative stress and inflammatory signals precipitates a change in astroglial function. Glial indoleamine 2,3-dioxygenase (a tryptophan metabolizing enzyme) is up-regulated, resulting in greater production of neurotoxic kynurenine metabolites and quinolinic acid (QA) (102, 209, 212, 213). Altered astroglia diminish their neurotrophic production (including BDNF and GDNF – glial cell line-derived neurotrophic factor) and start extruding inflammatory cytokines and glutamate. Glutamate released from astroglia accesses extra-synaptic *N*-methyl-d-aspartate (NMDA) receptors, causing suppression of BDNF synthesis and activation of the proapoptotic cascade. QA is a potent NMDA agonist that may further potentiate excitotoxicity (209, 212). Furthermore, proinflammatory cytokines increase the expression of 5HT and dopamine transporters, further disrupting monoamine signaling (207, 209). Increased oxidative stress may further compromise monoamine synthesis by depleting BH4 (tetrahydrobiopterin), a key coenzyme in monoamine synthesis (207, 213). Elevated CSF IL-6 in a group of suicidal mood-disordered patients that included several bipolar subjects correlated with more rapid 5HT and dopamine turnover, as evidenced by increased levels of their metabolites (214). Less efficient monoamine signaling was correlated with higher levels of IL-6 and reflected in greater severity of depressive symptoms (214). Finally, a recent imaging study reported a correlation between increased expression of inflammatory genes and a greater hemodynamic response to emotional stimuli in vmPFC, amygdala, and hippocampus of mood-disordered patients (the group included eight bipolar subjects) relative to healthy controls. In the same study, elevated expression of inflammatory genes was also linked with decreased thickness of the subgenual ACC, hippocampus, and caudate in the mood-disordered group (213). In summary, immune dysregulation in bipolar disorder is associated with alterations in monoamine and glutamate signaling, impaired neuroplasticity and neurotrophic support, and changes in glial and neuronal function, most likely contributing to the symptomatic expression and medical comorbidities of this mood disorder.

## Changes in Neuroplasticity and Neurotrophin Signaling

The role of BDNF in mood disorders has received more attention than other members of the neurotrophin family. It is involved in neuronal maturation, differentiation and survival, synaptic plasticity, and long-term memory consolidation (215). Furthermore, compelling preclinical evidence suggests that BDNF plays an important role in regulating the release of serotonin, glutamate, and gamma-aminobutyric acid (GABA), as well as in slow-wave sleep modulation (216, 217). BDNF expression is particularly high in the cerebral cortex and hippocampus (215).

Evidence suggests that stress and excessive, inadequately regulated glucocorticoid signaling may interfere with hippocampal neurogenesis in the context of bipolar illness (218). The hippocampus plays an important role in the inhibitory regulation of the HPA axis; therefore, impairment in its plasticity may have a relevant role in the pathophysiology of bipolar disorder. Individuals endowed with at-risk alleles of the BDNF gene may have compromised ability to normalize HPA axis activity, thereby adding to mood-disorder pathology (219). In addition to its role in regulating the neuroplastic processes, BDNF also acts as a resilience factor, assisting the maturation and differentiation of the nerve cell progenitors (220). Furthermore, BDNF even acts as an immunomodulator in the periphery of the body (221). There seems to be a bidirectional communication between the immune system and neuroplasticity regulators. In fact, recent preclinical research has identified microglia-originated BDNF as a key contributor to neuronal tropomyosin-receptor-kinase-B (TrkB) (a BDNF receptor) phosphorylation and ensuing changes in synaptic plasticity. Thus, microglia BDNF release appears to have a central role in learning and memory-related synaptic plasticity (222). BDNF is released from neurons in two forms: as pro-BDNF (pBDNF), and its chemically abbreviated version, mature BDNF (mBDNF). These two molecules (pBDNF and mBDNF) participate in opposing functions: pBDNF binds to p75 receptor, initiating apoptosis, or shriveling of neurons, whereas, mBDNF has primary affinity for the TrkB receptor, which mediates neuroplasticity and resilience (223, 224). There are three different alleles of the gene regulating BDNF synthesis, depending on the valine to methionine substitution at position 66 of the pro-domain: val66met, val66val, and met66met. Met variants are accompanied by decreased BDNF distribution in the dendrites and impairment in regulated secretion, and are considered to be “vulnerability” alleles in mood disorders (223). Genetic studies have implicated this BDNF gene polymorphism in the risk for bipolar disorder, early disease onset, rapid cycling, suicidality associated with mood episodes, and treatment response (225–228). Several clinical studies have demonstrated decreased levels of BDNF in bipolar depressed and manic patients (215). Low levels of BDNF were correlated with clinical severity of depression and mania (229, 230). Although diminished BDNF levels were reported in both treated and untreated bipolar subjects, one study found normal levels of BDNF in euthymic, treated patients, suggesting a potential neurotrophic benefit of pharmacotherapy (215, 229). A reciprocal relationship between BDNF and inflammatory mediators is an important marker of the progression of bipolar disease. Chronicity of bipolar illness, repeated episodes, and aging, all have a synergistic impact on a decline of neurotrophic signaling and increase in inflammation (215, 231–234). In the later stages of bipolar disorder, an imbalance between inflammatory cytokines (especially TNF-alpha), mediators of oxidative stress, and BDNF persists even between episodes and is associated with metabolic disruption, progression of structural brain changes, and neurocognitive decline (85, 215, 231, 235). In fact, some authors associate progression in bipolar disorder-related cognitive decline with a greater reduction in BDNF signaling (79). However, there are several areas regarding the role of BDNF in bipolar disorder that need further clarification. We are not to misconstrue elevated BDNF signaling as a universally positive sign. BDNF increases in the nucleus accumbens and amygdala may be associated with addictive behaviors and negative affect, respectively (236). Therefore, better determination of the aberrant regional BDNF distribution in the brains of bipolar patients, as well as understanding the dynamic of BDNF fluctuations as they relate to bipolar symptoms and treatment response may provide us with clinically useful information. Moreover, we need to further elucidate the relationship between peripheral and central BDNF levels, as well as altered dynamics between proBDNF and mBDNF in the context of bipolar illness.

Moreover, serum neurotrophin-3, neurotrophin-4/5, and GDNF are also altered in bipolar disorder (215). GDNF is an important regulator of neuroplasticity, monoamine and GABA signaling, and microglia activation (237–240). Findings of studies investigating peripheral GDNF levels in bipolar disorder are less consistent than those in BDNF research. Euthymic bipolar patients were reported to have either similar or elevated GDNF levels compared with healthy controls (237, 241). Although some studies detected reduced GDNF levels in manic patients that correlated with symptom severity, others found elevated GDNF in mania (237, 241). Unlike depressive episodes in MDD, which tend to be associated with a reduction of GDNF, bipolar depression does not seem to influence GDNF levels (237). A recent randomized controlled trial evaluated the impact of treatment on serum GDNF levels in bipolar patients. At baseline serum GDNF concentrations were reduced in both medication-free manic and depressed bipolar patients, compared to the control group. After 8-weeks of therapy with mood-stabilizers and antipsychotics, remitted bipolar patients had similar GDNF serum levels as healthy controls, indicating a “normalizing” effect of the successful pharmacological treatment (240). Differences in methodology, patients’ age, and medication status may explain some of the discrepancies in outcomes of GDNF studies in bipolar disorder. Additionally, variability in GDNF findings may be a reflection of a biological heterogeneity within bipolar disorder. Given the importance of glial pathology in the pathogenesis of bipolar disorder and the key role played by GDNF in stabilizing microglia activation and the propagation of peripheral inflammatory signaling in the CNS, we hope that future, methodologically rigorous studies may elucidate its disease state-dependent changes.

## Alterations in GABA, Glutamate, and Monoamine Transmission

Early research into the role of monoamine disturbances in bipolar disorder followed the path set by MDD studies. A study that included a mix of MDD and bipolar depressed patients noted an association between elevated CSF levels of 3-methoxy-4-hydroxyphenylglycol (MHPG), a norepinephrine metabolite, and agitation and anxiety in depressed patients (242). Additionally, studies reported diminished immunoreactivity of locus coeruleus processes and decreased CSF MHPG in suicidal bipolar subjects compared with controls (243, 244).

A recent review utilized cumulative pharmacological and imaging evidence to put forth the hypothesis of dopaminergic dysfunction in bipolar illness. This idea posits that excessive dopaminergic activity in the course of mania precipitates dopamine receptor down-regulation, which subsequently triggers a transition into a depressed state (245). Moreover, studies linking the severity of bipolar symptoms to tardive dyskinesia, even in the absence of pharmacotherapy, lend further support to claims of dopamine dysfunction in this disease state (246). Unfortunately, definitive and more direct evidence implicating dopamine in the etiology of bipolar disorder is still unavailable.

Brain imaging data implicating serotonin transporter (5HTT) binding in the pathophysiology of bipolar disorder are mixed, at best. An initial study of depressed, unmedicated bipolar patients reported increased 5HTT binding in the thalamus, dorsal ACC, medial PFC, and insula, and decreased binding in serotoninergic brainstem nuclei raphe compared with controls (247). A subsequent study noted reduced 5HTT binding in midbrain, amygdala, hippocampus, thalamus, putamen, and ACC of unmedicated bipolar depressed subjects compared with matched controls (248). Finally, a case report of a patient experiencing mixed mania, utilizing single-photon emission computed tomography imaging, detected elevated 5HTT binding in the midbrain and dopamine transporter binding in the striatum, which normalized after a year of psychotherapy (249). In conclusion, evidence of monoamine involvement in the etiology of bipolar disorder is for the most part indirect, inconsistent, and lacking replication in larger scale studies.

Relatively few studies have focused on abnormalities of GABA transmission in bipolar disorder. Recent studies have reported significantly increased GABA platelet uptake in bipolar depressed patients and decreased GABA uptake during mania (250). By contrast, glutamate platelet uptake was increased in the course of manic episodes relative to healthy controls. Altered platelet GABA and glutamate uptake correlated with the severity of depression and mania, respectively, as measured by standardized scales (250). Another study in euthymic bipolar patients, using magnetic resonance spectroscopy noted an increase in the GABA/creatinine ratio compared with healthy controls (251). Researchers have attempted to differentiate GABA transmission in unipolar depressed from bipolar depressed patients by analyzing glutamic acid decarboxylase (GAD) immunoreactive (ir) neuropil. GAD is the key enzyme in GABA synthesis. Unipolar depressed patients had a greater density of GADir neuropil in lateral dorsal thalamic nuclei, whereas bipolar depressed patients manifested a GADir decrease in dorsolateral PFC, compared with patients with MDD and healthy controls (252). This study suggests significant regional differences in GABA transmission between unipolar and bipolar depression.

Research into the expression of genes related to ionotropic glutamate receptors in bipolar disorder is relatively consistent, especially in relation to hippocampal glutamatergic abnormalities (253, 254). Studies noted a significant decrease in expression of the NR1 and NR2A subunits of NMDA glutamate receptors in the hippocampus and a significant increase in the expression of vesicular glutamate transporter-1 (VGluT1) in the ACC of bipolar subjects relative to a control group (255, 256).

An ongoing molecular dialog between glial cells and neurons has an important role in the regulation of glutamate signaling. Glutamate released from neurons is taken up by glial cells and converted to glutamine before being returned to neurons as the “raw material” for further neurotransmitter synthesis (257). In certain circumstances, astroglial cells are also capable of glutamate release (258, 259). Neuronal NMDA receptors respond to these astrocytic signals by elevating excitatory glutamatergic transmission (260). Reduction in both neuronal NMDA activity and astrocytic kainate receptor-mediated glutamate signaling by mood-stabilizing agents has been noted to have antidepressant activity in bipolar disorder (261). An MRS study has indicated an elevation in glutamine/glutamate ratio in the ACC and parieto-occipital cortex of manic subjects compared with matched controls, pointing to excessive glutamatergic activity and/or aberrant glial/neuronal interactions in the context of bipolar illness (257).

Several MRS studies and a postmortem study have reported an increase in glutamatergic transmission in the frontal cortex and hippocampus of bipolar subjects relative to control groups (262–264). Interpretation of MRS findings requires caution, however, because brain glutamate has functions other than neurotransmission. Proton magnetic resonance spectroscopy (^1^H MRS) provides an opportunity for *in vivo* evaluation of glutamate-related metabolites, depending on field strength and signal-to-noise ratio, glutamate and glutamine can be quantified either separately or as a composite of glutamate, glutamine, GABA, and other metabolites (most often labeled as Glx) (265). Aside from being the most plentiful neurotransmitter in the brain, glutamate is also a substrate in protein metabolism, and a precursor for glutamine, GABA, and glutathione. Unfortunately, MRS signals are of little aid of differentiating synaptic glutamate from the intracellular glutamate-related compounds (266). Multiple storage locations, difficulty discerning glutamate-related metabolites from each other and their diverse roles makes precise interpretation of MRS Glx signal a daunting challenge. However, since GABA represents only a minor fraction, one can reasonably approximate Glx as a representative of combined glutamine and glutamate, 80% of which is stored in synaptic vesicles, while 20% is in adjacent astrocytes awaiting conversion to glutamine (266).

Recent comprehensive meta-analyses have identified relatively consistent (albeit with a few discrepant findings) elevation of Glx in ACC, medial PFC, DLPFC, parieto-occipital cortex, insula, and hippocampus. These findings persisted across the bipolar mood states and even in euthymic bipolar patients, relative to the control group (265). Effect sizes related to Glx signal were more robust in mania and depression than in euthymic patients (266). One can speculate that at least some of the glutamatergic abnormality in bipolar disorder reflects functional and numerical glial abnormalities given their cardinal role in regulation of glutamate metabolism and signaling (266). Distribution of aberrant Glx signals in bipolar disorder also substantially overlaps with glial alterations reported in the post-mortem cytological studies. Anatomical structures characterized by anomalous MRS signals in bipolar disorder are some of the key components of the cortico-limbic regulatory pathways, involved in regulation of mood, cognitive processing, autonomic, and endocrine response. It would be plausible to assume that altered glutaminergic signaling in these principal cortico-limbic circuits may be reflected in diverse bipolar clinical symptomatology.

Glutamatergic findings in bipolar disorder were similar whether the patients were medicated or not. Interestingly, one of the studies demonstrated an inverse relationship between diurnal salivary cortisol levels and hippocampal glutamate concentration in bipolar patients (262). This finding reaffirms a critical link between neuroendocrine disturbance and glutamate transmission in bipolar disorder, implicating this key area involved in memory, emotional regulation, and stress response.

Overall, multiple, consistent, and convergent evidence from genetic, postmortem, biochemical, and imaging studies points to a principal role of glutamatergic dysregulation in the etiopathogenesis of bipolar disorder. Moreover, evidence links aberrant glial–neuron interactions and endocrine dysregulation with alterations in glutamatergic transmission.

## Changes in the Intracellular Signaling Cascades

It is becoming increasingly evident that current mood-stabilizing agents have actions that extend beyond binding to neuronal membrane surface receptors. Therapeutic actions of psychotropics utilized in the treatment of bipolar disorder most likely rely on an interface with intracellular signaling cascades and eventual enduring changes in gene expression, accompanied by alterations in neurotransmission and neuroplasticity. Better understanding of intracellular signaling cascades may therefore provide valuable insights into the underlying causes of bipolar disorder and subsequently to more effective treatment strategies.

The phosphoinositide-3-kinase (PI3K)/AKT pathway is a general signal transduction pathway for growth factors, including BDNF and consequently for BCL-2. The GSK-3 signaling pathway modulates apoptosis and synaptic plasticity. Increased activity in the GSK-3 pathway supports apoptosis. Attenuation of GSK-3 activity leads to up-regulation of BCL-2 and beta-catenin and consequent enhancement of neuroplasticity and cellular resilience. This pathway is also involved in circadian regulation (60, 61).

Interestingly, manipulation of the GSK-3 pathway produces both antimanic and antidepressant effects. Many agents with mood-stabilizing properties, such as lithium, valproate, and atypical antipsychotics, directly and indirectly modulate the PI3K, GSK-3, and Wnt signaling pathways, the very same ones implicated in genetic studies of bipolar disorder (267, 268).

There is another surprising outcome from genetic studies of bipolar disorder: the affected stress-activated kinase pathways do not target neurotransmitter trafficking; they are funneled toward regulating oligodendroglia (61). Thus, the convergence of genetic vulnerabilities in bipolar disorder appears to particularly target oligodendrocyte function (61), inviting speculation about the role of stress and circadian dysregulation in precipitating white matter changes in cortico-limbic pathways, which are critical for proper mood regulation.

## Changes in Synaptic Function, Bioenergetics, and Oxidative Metabolism

Convergent evidence from imaging, neurochemical, and genetic studies points to disturbances in bioenergetics and mitochondrial function in the context of bipolar illness (268, 269). A substantial portion of genes implicated in the etiology of bipolar disorder code for mitochondrial proteins. Hippocampal expression of genes related to mitochondrial proteins was substantially reduced in bipolar compared with control subjects (270). Previously described disease-related functional alterations in brain circuitry may have a reciprocal relationship with mitochondrial function. Namely, genetic control of mitochondrial function is influenced by the level of neuronal activity (268).

Beyond its well-known role in cellular bioenergetics, proper mitochondrial function is important for the regulation of neuroplasticity, apoptosis, and intracellular calcium levels. Of course, dynamic changes in endocellular calcium have a crucial role in the modulation of intracellular signaling cascades and neurotransmitter release (271). Furthermore, compromised mitochondrial function may be reflected in aberrant oxidative metabolism, down-regulated adenosine triphosphate-dependent proteasome degradation, and ensuing DNA damage contributing to neuronal apoptosis (270, 272).

The accelerated telomere shortening found in bipolar disorder may also be a consequence of stress-related oxidative damage. A recent study suggested that telomere shortening in mood disorders, most likely attributable to oxidative stress, may be equivalent to 10 years of accelerated aging (273).

## Integration of Neurobiological Findings

As we noted at the beginning of this review, from a neurobiological perspective, there is no such thing as bipolar disorder. Rather, it is almost certainly the case that there are many somewhat similar, but subtly different, pathological conditions that produce a final common pathway disease state that we currently diagnose as bipolarity. This heterogeneity – reflected in the lack of synergy between our current diagnostic schema and our rapidly advancing scientific understanding of the condition – puts a hard limit on all attempts to articulate an integrated perspective on bipolar disorder. Also posing a challenge to the integrative enterprise is the fact that nothing could be further from a static condition than bipolar disorder. Whereas, most psychiatric conditions vacillate within a single register between symptom exacerbation and various degrees of recovery, those attempting to fully understand bipolar disorder must contend with the fact that exacerbations come in two distinct flavors – manias and depressions – and that often these exacerbations can take any of a nearly infinite number of combinations of these two mood disturbances.

Despite these challenges, scientific findings in recent years are beginning for the first time to offer a provisional “unified field theory” of the disease. The very fact that no single gene, pathway, or brain abnormality is likely to ever account for the condition is itself an extremely important first step in better articulating an integrated perspective on both its ontological status and pathogenesis. Whether, this perspective will translate into the discovery of innumerable more homogeneous forms of bipolarity is one of the great questions facing the field and one that is likely to have profound treatment implications, given the fact that such a discovery would greatly increase our ability to individualize – and by extension, enhance – treatment.

It is intriguing that despite the primacy given to functional neuroimaging methodologies in current psychiatric research, results from fMRI are among the least consistent in the context of bipolar disorder in terms of separating bipolar patients from healthy controls and from other psychiatric conditions, as well as for differentiating mania from depression. More consistent findings have emerged at a cellular level, providing evidence that bipolar disorder is reliably associated with dysregulation of glial–neuronal interactions and with abnormalities more apparent in glial elements than in neurons. Among these glial elements are microglia – the brain’s primary immune elements, which appear to be overactive in the context of bipolarity. Multiple studies now indicate that inflammation is also increased in the periphery of the body in both the depressive and manic phases of the illness, with at least some return to normality in the euthymic state. These findings are consistent with changes in the HPA axis, such as reduced sensitivity to glucocorticoids, which are known to drive inflammatory activation.

Further evidence that classification schemes based on the science of the future will share only minimal overlap with our current diagnostic categories comes from recent data in genetics, neuroscience, and immunology demonstrating that bipolar disorder shares many features with other conditions, especially schizophrenia and unipolar depression, which we currently conceptualize as separate disease states. Recent genetic studies identify many risk loci shared by these conditions. Although some of these risk alleles primarily target CNS functioning, many others have far more basic “housekeeping” functions within most cells in the body. These findings provide a novel insight into other recent discoveries linking bipolar disorder to abnormalities in metabolism and general and mitochondrial function in particular. Moreover, the fact that bipolarity reaches so deeply into the core processes of life itself may enrich our understanding of why the disorder is so reliably associated with immune abnormalities and with a marked escalation in risk for the development of multiple medical conditions that account for much of the increased mortality associated with the disorder.

Given the dynamic nature of interactions among microglia, astroglia, and oligodendroglia – all of which influence synaptic activities essential to mental functioning – it should perhaps not be surprising that bipolar disorder in particular, and psychiatric conditions in general, remain hard to characterize using monolithic diagnostic, or even physiological, criteria. Adding to the complexity inherent in glial–neuronal interactions is the fact that these interactions are further influenced by neural transmission, as well as immune, endocrine, and neurotrophic signaling. The sum of these signals affects intracellular signaling cascades which, in turn, initiate changes in gene expression that initially cause functional changes that over time alter the very structure of the brain itself. As a result of disrupted homeostatic functioning at multiple levels ranging from the molecular to the environmental, one maladaptive change begets another in bipolar disorder. Macroscopic alterations drive microscopic ones, and vice versa.

In this review, we have emphasized the complexity of bipolar illness, not just because this is what current science suggests, but also because this perspective implies a need for parallel dynamic changes in the ways we diagnose and treat the condition. For example, bipolar disorder will not look the same in teenage years, adulthood, and senescence. As our scientific understanding advances, we suspect that we will gain greater understanding of how the ever-changing nature of the disease process requires different combinations of therapeutic interventions, with these treatment modalities tracking changes in the substrate and pathophysiological mechanisms of the disease in an iterative manner. Said differently, to significantly advance how we treat bipolar disorder, we will need to replace unidimensional (i.e., purely phenomenological diagnoses) and static (i.e., based on the assumption that the appearance and underlying pathophysiology of the disease do not evolve over time) diagnostic and treatment approaches with strategies that are dynamic and integrated (i.e., including elements such as psychotherapy, pharmacotherapy, exercise, nutrition, meditation, relationship healing, etc.) as well as multi-level (i.e., based on phenomenology, neuroimaging, and biochemical and genetic evaluation).

## Author Contributions

Vladimir Maletic was responsible for the design, research, and writing of this article. Charles Raison contributed to the design and writing of this article.

## Conflict of Interest Statement

Dr. Vladimir Maletic has served on advisory boards for Eli Lilly and Company, Lundbeck, Otsuka America Pharmaceuticals, Inc., Pamlab, Pfizer, Sunovion, Teva Pharmaceuticals, and Takeda Pharmaceuticals and as a speaker for Eli Lilly and Company, Lundbeck, Merck, Pamlab, Pfizer, Sunovion, Teva Pharmaceuticals, and Takeda Pharmaceuticals, and has prepared CME materials for NACCME and CME Incite. Dr. Charles Raison has served on advisory boards for Lilly and Pamlab and as a speaker for Pamlab and has prepared CME materials for NACCME and CME Incite.
